# Determinants of birth asphyxia among preterm newborns in Ethiopia: a systematic review and meta-analysis of observational studies protocol

**DOI:** 10.1186/s13643-022-01905-8

**Published:** 2022-02-19

**Authors:** Abay Woday Tadesse, Muluken Dessalegn Muluneh, Setognal Birara Aychiluhm, Kusse Urmale Mare, Gebeyaw Biset Wagaw

**Affiliations:** 1 College of Medicine and Health Sciences, Samara University, Samara, Afar Region Ethiopia; 2 Dream Science and Technology College, Dessie, Ethiopia; 3 Armauer Hansen Research Institute, Addis Ababa, Ethiopia; 4 Amref Health Africa, Addis Ababa, Ethiopia; 5 Western Sydney University, Sydney, Australia; 6 College of Medicine and Health Sciences, Wollo University, Dessie, Amhara Region Ethiopia

**Keywords:** Preterm Newborns, Meta-analysis, Birth asphyxia, Difficulty of breathing at birth, Determinants, Ethiopia

## Abstract

**Background:**

Birth asphyxia accounted for nearly 50% of neonatal mortality in Sub-Saharan African countries. This scenario has been worst in Ethiopia where every two out of three deaths attributed to birth asphyxia. Moreover, studies conducted in Ethiopia were highly variable and inconclusive to estimate the pooled prevalence and determinants of perinatal birth asphyxia among preterm babies.

**Objective:**

This study aimed to estimate the pooled prevalence of birth asphyxia and its determinants among preterm newborns in Ethiopia.

**Methods:**

The protocol for this review is registered at PROSPERO with registration number CRD42020158224. A comprehensive online databases (PubMed, HINARI, Scopus, EMBASE, Science direct, and Cochrane library database), Google Scholar, African Journals online, other gray and online repository accessed studies will be searched using different search engines. In addition, maternity and infant care databases uploaded at Ethiopian Health Development Journal and Ethiopian Journal of Health Sciences will be searched until 30 June 2020. Newcastle-Ottawa Quality Assessment Scale (NOS) will be used for critical appraisal of studies. Three reviewers will screen all retrieved articles, conduct data extraction, and then critically appraise all identified studies. All identified observational studies reporting the prevalence of birth asphyxia and associated factors among neonates in Ethiopia will be considered. The analysis of data will be done using STATA 11.0. We will demonstrate pooled estimates and determinants of birth asphyxia with effect size and 95% confidence interval. Heterogeneity among the included studies will be assessed through the Cochrane *Q* test statistics and *I*^2^ test. Publication bias will be checked using funnel plot and Egger’s test. Finally, statistical significance level will be declared at a *p* value of less than 0.05.

**Discussion:**

The result from this systematic review will inform and guide health policy planners to invest limited resources on maternal and neonatal health. Furthermore, it will be a stimulus for future cumulative meta-analysis researchers in developing nations.

**Supplementary Information:**

The online version contains supplementary material available at 10.1186/s13643-022-01905-8.

## Background

The World Health Organization (WHO) defines birth asphyxia as failure to initiate and sustain normal breathing at the first and fifth minute of birth [1]. Although more than two-thirds of newborn deaths are preventable, most of the neonatal deaths occur at home, uncounted, and invisible to national and regional policies and programs [2]. The prevalence of birth asphyxia varies across the globe and African countries contribute nearly 50% of the total magnitude of birth asphyxia [3–6]. Similarly, in Sub-Saharan countries, the prevalence of birth asphyxia is still unacceptably high which ranges from 21.3 to 56.9% [4, 7–11]. In Ethiopia, the magnitude of birth asphyxia varies between 3.1% and 56.9% in Ethiopia [10–13].

Globally, 2.5 million children and more than a million African babies are estimated to die in the first month of life, annually [14]. Worldwide, 25% of all neonatal deaths are attributed to birth asphyxia [15]. In Ethiopia, two-thirds of neonatal deaths are attributed to birth asphyxia [16]. Furthermore, birth asphyxia is the first cause of neonatal deaths (31.6%) followed by prematurity (21.8%) and sepsis (18.5%) in developing countries, like Ethiopia [17].

Studies conducted across the globe have identified factors associated with birth asphyxia. These include antepartum risk factors (i.e., maternal age, maternal education, pre-eclampsia, primi-gravidity) intrapartum risk factors (i.e., prolonged labor, premature rupture of membrane, non-cephalic presentation, mode of delivery, and maternal fever), and fetal risk factors (i.e., pre-term babies, fetal distress, and baby weight) [18–22].

Globally and regionally, various strategies and interventions have been implemented to decline neonatal mortality rates because of birth asphyxia [14, 15, 23]. For instance, one of the main objective in the Sustainable Development Goals of 2030 (SDG-3) is to reduce the neonatal mortality rate below 12 deaths per 1000 live births [24]. Similarly, Ethiopia has made remarkable progress in achieving many of the national child health indicators [25] and it has designed the National Child Survival Strategy to reduce child mortality [26]. However, the burden of birth asphyxia is still unacceptably high in the country. There was a single review study conducted in Central Africa to estimate the pooled prevalence of perinatal birth asphyxia among newborns babies [27]. However, this study does not separately assess the current situation of Ethiopia regarding birth asphyxia among preterm babies. Furthermore, the previous studies conducted in different parts of Ethiopia were highly inconsistence and inconclusive. Moreover, none of these studies has shown the pooled estimates of birth asphyxia and its determinants that will help the policy makers to develop appropriate preventive mechanisms for the country. Thus, central aim of this systematic review and meta-analysis is to have pooled estimate of birth asphyxia and its determinants among preterm newborns in Ethiopia.

## Objective

### General objective

To estimate the pooled prevalence and associated factors of birth asphyxia among preterm newborns in Ethiopia.

### Specific objectives

- To determine the pooled prevalence of birth asphyxia among preterm newborns in Ethiopia
- To identify pooled effect size for determinants of birth asphyxia among preterm newborns in Ethiopia.

## Research questions

1. What is the estimated pooled prevalence of birth asphyxia among preterm newborns in Ethiopia?
2. What are the determinants of birth asphyxia among preterm newborns in Ethiopia?

## Methods and materials

### Search strategy

The protocol for this review was registered at PROSPERO with registration number CRD42020158224 [https://www.crd.york.ac.uk/prospero/#recordDetails]. We will apply the Preferred Reporting Items for Systematic review and Meta-analyses (PRISMA-2009) diagram to show the screening process of the included studies and (PRISMA-P 2015) (Additional file 1) [28] statement to present the findings of the study. Different Online databases (PubMed, HINARI, Scopus, EMBASE, Science direct, Cochrane library database, and African Journals online), and other gray literatures like Google Scholar and extended referencing of already searched articles will be used to search articles on birth asphyxia. Searching terms were developed based on the formulated research questions during protocol development. We have developed different Boolean operators to have comprehensive database searches on birth asphyxia. Thus, the international online electronic database repositories include; “perinatal asphyxia” OR “birth asphyxia,” OR “breathing difficulty at birth,” OR “neonatal asphyxia,” OR “low APGAR score,” OR “hypoxic-ischemic encephalopathy” OR “Neonatal Encephalopathy” AND “determinants” OR “predictors,” OR “risk factors,” OR associated factors,” OR causes,” AND “newborn,” OR neonate,” AND “Ethiopia” (Additional file 2).

Moreover, we will utilize snowballing to screen the references of identified articles for potentially relevant studies. Furthermore, studies identified by our database searching strategy will be retrieved and managed using Endnote X8 (Thomson Reuters, Philadelphia, PA, USA) software.

### Eligibility criteria

#### Inclusion criteria

##### Setting/context

This review will include studies conducted in Ethiopia.

##### Population

The review will include studies involving newborns (preterm, near-term, term, and post-term newborns) immediately after birth (1–5 min of birth) and the babies who were born after 28 weeks of completed gestations (after the age of viability).

##### Study design

All observational studies (i.e., cross-sectional, case-control, and cohort) that have reported the prevalence of birth asphyxia and its determinants among newborns.

##### Language

Studies reported in the English language will be considered.

##### Publication year

No restriction on year of publication.

#### Exclusion criteria

We will exclude studies published in languages other than English. In addition, we will exclude studies other than observational studies like, case reports, conference reports, national survey reports, and expert opinions that have not precisely measured both the prevalence and the determinants of birth asphyxia among preterm newborns.

### Outcome measurement

This systematic review and meta-analysis had two main outcomes. The first outcome was the prevalence of birth asphyxia among the neonates. In this study, birth asphyxia was defined as newborn with Apgar score less than 7 at the 1st or 5th minutes of birth. The pooled prevalence was calculated by dividing the number of neonates with birth asphyxia to the total number of neonates who have been included in the study (total sample size) multiplied by 100. The second outcome of the study was to identify maternal and neonatal factors associated with birth asphyxia that was determined in the form of pooled odds ratio. Moreover, the outcomes of this study were to focus on single studies estimating the prevalence and associated factors of birth asphyxia among live newborns in Ethiopia.

### Selection of studies

Two authors (AWT and GBW) will review the studies based on inclusion and exclusion criteria. The review will follow two stages. In the first stage, reviewers will assess both the titles and abstracts of the studies identified from the search. In the second stage, full-text screening will done to screen the full texts selected in the previous stage. The extended reference searching of included studies will be considered for those articles not accessible using online databases. If the articles are not open access, we will contact the corresponding author at least for three times. If the authors are not willing to provide the full text, we will exclude that specific article.

We will provide reason for exclusion for all excluded studies in the PRISMA-2009 flow diagram. Finally, we will prepare a final list of articles for data extraction

### Data extraction

Three authors (AWT, SBA, and GBW) will independently extract all necessary data using a standardized data extraction format (Additional file 3). We will pretest the data extraction form on three studies of each type, to ensure that it adequately facilitates the collection of all necessary data required for an effective systematic review and meta-analysis. Discrepancies between data extractors will be discussed to reach consensus. If a consensus cannot be reached, the authors will consult a third reviewer (KUM). For each included articles, we will record the first author’s last name, year of publication, the setting where the study was conducted, study design, study period, sample size, the response rate, the population, outcome definition, comparison groups, and the effect estimate.

### Quality assessments

Four authors (AWT, SB, KU, and GB) will independently conduct quality assessment of included studies. Newcastle-Ottawa Quality Assessment Scale (NOS) for observational studies (i.e. cross-sectional, case-control, and cohort) was employed [29, 30] will be used to assess the quality of a study and to determine the extent to which a study addressed the possibility of bias in its design, conduct, and analysis (Additional file 4). In customising the scale to fit this study, we will took into account the study sampling methods and similarities between the study groups regarding adjustment for confounding factors, the ascertainment of exposure and outcomes, and study design. The abstracting tool will include different questions based on the study designs. The four investigators independently will perform the quality assessment while abstracting the data for the meta-analysis. The quality scores of the four abstractors will be averaged. Finally, the studies with NOS score of six and more were will be considered as a “good” quality study (low risk) while studies scored less than six will be considered as a “poor” quality study (high risk) [30].

### Data synthesis and analysis

The extracted data will be entered into a Microsoft Excel Database and then imported into STATA version 14.0 (Stata Corp. LLC, TX, USA) software with packages of meta-analysis for further analysis. The researchers will perform a narrative description of the study population, the characteristics of included studies, number of included studies, number of participants from the included studies, the risk factors identified, and the cause for birth asphyxia as well as the outcome characteristics. We will use PRISMA-2009 flow diagram to show the screening stage of included and reason for excluded studies. We will present the descriptive sections using texts, tables, and figures to summarize the selected studies and results.

The pooled prevalence of birth asphyxia in Ethiopia will be demonstrated. We will use fixed effect model if the studies have similar methodology, same population, and study design. If not, we will utilize the random effect model [31] to handle heterogeneity between studies. Then, the “metaprop” command with random-effects models at 95% confidence intervals will be used to estimate the pooled prevalence of birth asphyxia at national level [32]. The Freeman Tuckey variant of the arcsine square root transformation of proportions will be fitted to avoid variance instability when handling proportions close to one [33]. We will assess heterogeneity by using chi-squared test on Cochran’s *Q* statistic with a 5% level of statistical significance [26] and *I*^2^ statistic test [34], assuming that *I*^2^ value of 25%, 50%, and 75% being representative of low, moderate, and high heterogeneity, respectively [34]. If the heterogeneity is significant (*I*^2^ > 75%), then, we will conduct subgroup analyses and meta-regression to investigate sources of heterogeneity.

Publication bias will be examined by the visual inspection of funnel plots [35] and Egger’s test [36]. A *p* value < 0.10 will be considered indicative of statistically significant publication bias. Thus, if there is evidence of publication bias, we will use Duval and Tweedie’s trim-and-fill method [37].

For factors associated with birth asphyxia, two-by-two tables will be constructed (if possible), the odds ratio with 95% confidence interval will be calculated. Then, the statistically significance level will be declared at a *p* value less than 0.05. However, if the meta-analysis is not possible, we will conduct narrative synthesis.

### Subgroup and sensitivity analyses

Sub-group analysis will be performed based on sample size, regions, year of publication, and quality of studies. Finally, to conduct sensitivity analysis, we will assess the stability or robustness of the pooled estimates to outliers and the impact of individual studies [37].

## Discussion

This review will provide a detailed summary of the evidence on the prevalence and determinants of birth asphyxia among preterm newborns in Ethiopia. This review will be the first evidence on the prevalence of birth asphyxia among Ethiopian preterm babies. The findings of this review will fill scarce of evidence to understand the prevalence and determinants of birth asphyxia among preterm babies in the country. Thus, the results from this review will inform health policy planners and researchers to have up-to-date information that will help them to develop appropriate action points to reduce burden of birth asphyxia in the country.

### Dissemination of results

The results of this systematic review and meta-analysis will be published in a peer-reviewed journal and presented at national and international research conferences.

## Supplementary Information

**Additional file 1.** PRISMA-P 2015 checklist.

**Additional file 2.** PubMed search string.

**Additional file 3.** Articles screening checklist.

**Additional file 4.** Adapted Newcastle-Ottawa. Quality Assessment Scale (NOS)

## Data Availability

All materials and data related to this article are included in the main document of the manuscript. However, if anyone has interested to have raw data, he/she can contact the corresponding author.

## Abbreviations

EDHS: Ethiopian Demographic and Health Survey
LMICs: Low- and middle-income countries
MeSH: Medical Subject Headings
NOS: Newcastle Ottawa Quality Assessment Scale
PRISMA: Preferred Reporting Items for Systematic Review and Meta-Analysis
WHO: World Health Organization
