# Supplementary material for: Determinants of birth asphyxia among preterm newborns in Ethiopia: a systematic review and meta-analysis of observational studies protocol
Source: Syst Rev. 2022 Feb 19;11:30. doi: 10.1186/s13643-022-01905-8 (PMC8858466; doi:10.1186/s13643-022-01905-8)
Supplement: Supplementary file 2 — Additional file 2. PubMed search string. [file 13643_2022_1905_MOESM2_ESM.docx]

## **Additional file 2**: **PubMed search string**

| Search | Query | Items found | Date | Time |
| --- | --- | --- | --- | --- |
| #2 | Search ((birth asphyxia OR perinatal asphyxia OR breathing difficulty OR low APGAR score) | 65563 | 5/9/2020 | 9:31:54 |
| #1 | Search ((birth asphyxia OR perinatal asphyxia OR breathing difficulty OR low APGAR score) AND (determinants OR predictors OR "risk factors" OR causes) | 38546 | 5/9/2020 | 9:30:40 |
| #1 and # 2 | Search (((birth asphyxia OR perinatal asphyxia OR breathing difficulty OR low APGAR score) AND (determinants OR predictors OR "risk factors" OR causes) AND (Ethiopia OR Ethio)) | 62 | 5/9/2020 | 9:27:53 |
