# Supplementary material for: Determinants of birth asphyxia among preterm newborns in Ethiopia: a systematic review and meta-analysis of observational studies protocol
Source: Syst Rev. 2022 Feb 19;11:30. doi: 10.1186/s13643-022-01905-8 (PMC8858466; doi:10.1186/s13643-022-01905-8)
Supplement: Supplementary file 3 — Additional file 3. Articles screening checklist. [file 13643_2022_1905_MOESM3_ESM.docx]

## **Additional file 3: Articles screening checklist**

| **ID** | **List of author** | **Publication year** | **Region** | **Study design** | **Sample size** | **Event** | **Quality score** | **Objective of study** |
| --- | --- | --- | --- | --- | --- | --- | --- | --- |
| 1 |  |  |  |  |  |  |  |  |
| 2 |  |  |  |  |  |  |  |  |
| 3 |  |  |  |  |  |  |  |  |
| 4 |  |  |  |  |  |  |  |  |
| 5 |  |  |  |  |  |  |  |  |
| 6 |  |  |  |  |  |  |  |  |
| 7 |  |  |  |  |  |  |  |  |
| 8 |  |  |  |  |  |  |  |  |
| 9 |  |  |  |  |  |  |  |  |
| 10 |  |  |  |  |  |  |  |  |
