# Supplementary material for: Determinants of birth asphyxia among preterm newborns in Ethiopia: a systematic review and meta-analysis of observational studies protocol
Source: Syst Rev. 2022 Feb 19;11:30. doi: 10.1186/s13643-022-01905-8 (PMC8858466; doi:10.1186/s13643-022-01905-8)
Supplement: Supplementary file 4 — Additional file 4. Adapted Newcastle-Ottawa. Quality Assessment Scale (NOS) [file 13643_2022_1905_MOESM4_ESM.docx]

## **Additional 4: Adapted Newcastle-Ottawa Quality Assessment Scale (NOS)**

NOS quality assessment tools available at:

1. <http://www.ncbi.nlm.nih.gov/pubmedhealth/PMH0049222/pdf/TOC.pdf>
2. <http://www.ohri.ca/programs/clinical_epidemiology/oxford.asp>

## **Quality appraisal checklist for observational studies**

| **ID** | **List of Authors** | **Publication year** | **Methods & Materials (4pts)** | **Comparability (2pts)** | **Outcome/Exposure measurement & analysis (3pts)** | **Total (9pts)** | **Decision** |
| --- | --- | --- | --- | --- | --- | --- | --- |
| 1 |  |  |  |  |  |  |  |
| 2 |  |  |  |  |  |  |  |
| 3 |  |  |  |  |  |  |  |
| 4 |  |  |  |  |  |  |  |
| 5 |  |  |  |  |  |  |  |
| 6 |  |  |  |  |  |  |  |
| 7 |  |  |  |  |  |  |  |
| 8 |  |  |  |  |  |  |  |
| 9 |  |  |  |  |  |  |  |
| 10 |  |  |  |  |  |  |  |
| 11 |  |  |  |  |  |  |  |
| 12 |  |  |  |  |  |  |  |
| 13 |  |  |  |  |  |  |  |
| 14 |  |  |  |  |  |  |  |
| 15 |  |  |  |  |  |  |  |
| 16 |  |  |  |  |  |  |  |
| 17 |  |  |  |  |  |  |  |
| 18 |  |  |  |  |  |  |  |
| 19 |  |  |  |  |  |  |  |
| 20 |  |  |  |  |  |  |  |
| 21 |  |  |  |  |  |  |  |
